# Anisotropic modulation of magnetic properties and the memory effect in a wide-band (011)-Pr_0.7_Sr_0.3_MnO_3_/PMN-PT heterostructure

**DOI:** 10.1038/srep09668

**Published:** 2015-04-24

**Authors:** Ying-Ying Zhao, Jing Wang, Hao Kuang, Feng-Xia Hu, Yao Liu, Rong-Rong Wu, Xi-Xiang Zhang, Ji-Rong Sun, Bao-Gen Shen

**Affiliations:** 1 Beijing National Laboratory for Condensed Matter Physics and the State Key Laboratory of Magnetism, Institute of Physics, Chinese Academy of Sciences, Beijing 100190, P. R. China; 2 Physical Science and Engineering, King Abdullah Univ Sci & Technol, Thuwal 23955-6900, Saudi Arabia

## Abstract

Memory effect of electric-field control on magnetic behavior in magnetoelectric composite heterostructures has been a topic of interest for a long time. Although the piezostrain and its transfer across the interface of ferroelectric/ferromagnetic films are known to be important in realizing magnetoelectric coupling, the underlying mechanism for nonvolatile modulation of magnetic behaviors remains a challenge. Here, we report on the electric-field control of magnetic properties in wide-band (011)-Pr_0.7_Sr_0.3_MnO_3_/0.7Pb(Mg_1/3_Nb_2/3_)O_3_-0.3PbTiO_3_ heterostructures. By introducing an electric-field-induced in-plane anisotropic strain field during the cooling process from room temperature, we observe an in-plane anisotropic, nonvolatile modulation of magnetic properties in a wide-band Pr_0.7_Sr_0.3_MnO_3_ film at low temperatures. We attribute this anisotropic memory effect to the preferential seeding and growth of ferromagnetic (FM) domains under the anisotropic strain field. In addition, we find that the anisotropic, nonvolatile modulation of magnetic properties gradually diminishes as the temperature approaches FM transition, indicating that the nonvolatile memory effect is temperature dependent. By taking into account the competition between thermal energy and the potential barrier of the metastable magnetic state induced by the anisotropic strain field, this distinct memory effect is well explained, which provides a promising approach for designing novel electric-writing magnetic memories.

With the rapid increasing requirements for information storage, developing compact, innovative devices that offer fast, energy-efficient nonvolatile random access memory is becoming a significant and challenging task. To meet this challenge, a new way to control magnetism via electric fields^1^,^2^,^3^, using the converse magnetoelectric (ME) effect, rather than electric currents or magnetic fields, is attracting tremendous attention. Initial research suggests that the top candidates for realizing electric-field control of magnetism are single-phase multiferroic materials with simultaneous magnetic and ferroelectric orders; however, more recent experiments show that these materials have small converse ME effects and are unsuited to practical application^4^. As an alternative, artificial multiphase systems that consist of both ferromagnetic (FM) and ferroelectric (FE) materials are receiving more attention in recent years^1^,^2^,^3^. Coupling of the two ferroic phases suggests that an electric field may be able to control magnetic properties. Previous experimental work has demonstrated that an electric field can control magnetic anisotropy and remnant magnetization by using strain-mediated ME coupling in heterostructures with FM films grown on FE substrates such as epitaxial La_0.67_Sr_0.33_MnO_3_/BiTiO_3_^4^, La_2/3_Sr_1/3_MnO_3_/PMN-PT^5^, polycrystalline Ni/PMN-PT, Fe_3_O_4_/PZNPT, CoFe_2_O_4_/PMN-PT, and CoFe/BiFeO_3_^6^,^7^,^8^,^9^. For the most part, these studies used the linear-converse piezoelectric response to induce changes in magnetic anisotropy and remnant magnetization, which typically return to their initial state once the driving electric field is removed. However, a nonvolatile tuning of the magnetic state by the electric field, namely the memory effect, is required for information storage.

Perovskite manganites contain rich physical phenomena: the Jahn-Teller (JT) distortion, double-exchange coupling, metal-insulator transition, and phase separation due to the strong interplay among lattice, charge, spin, and orbital degrees of freedom. Meanwhile, the lattice strain can modulate most of these properties by modifying MnO_6_-octahedron distortion and thus the strength of the double-exchange interaction and the JT coupling^10^,^11^. Especially, due to the strong coupling between lattice and spin, an electric field is able to control the magnetic properties of manganite films through the converse piezoelectric effect or the polarizing effect of the FE substrate^2^. It is well accepted that heterostructures composed of piezoelectric oxide and manganite film are a promising platform for studying strain-mediated ME coupling, either for revealing fundamental principles or for exploring new functional devices. Recently, Chen et. al^12^ observed a nonvolatile memory effect of ME coupling/strain in narrow-band, phase-separated Pr_0.6_Ca_0.4_MnO_3_(PCMO)/PMN-PT heterostructures, and demonstrated that the electric-field control of magnetization is dominated by the change of phase separation in PCMO, which is nonvolatile and manifests a memory effect of ME coupling. It was assumed that such a nonvolatile magnetic memory effect was due to the modulated energy balance between coexisting FM and charge-ordered antiferromagnetic (COAFM) phases in the phase-separated manganite system.

Here, we report a memory effect of ME coupling in the heterostructure composed of a wide-band Pr_0.7_Sr_0.3_MnO_3_ (PSMO) manganite film grown on (011)-oriented 0.7Pb(Mg_1/3_Nb_2/3_)O_3_-0.3PbTiO_3_ (PMN-PT) substrate. Heterostructures were fabricated by using the pulsed-laser deposition (PLD) technique. The PSMO thin film was used as a model system because of its relatively large *e_g_* bandwidth with trivial phase separation and optimized metal-insulator/ferromagnetism-paramagnetism transition temperature^16^. In the absence of Sr doping, the Pr_0.7_Ca_0.3_MnO_3_ system has a narrow, one-electron e_g_ bandwidth and exhibits strong COAFM^13^. When Ca is successively substituted by Sr, the average ion radius is altered^14^ because Sr ions are larger, leading to an increase in electron bandwidth (W) and hence the hopping amplitude for electrons in the e_g_ band. Such an increase in W stabilizes the FM state by enhancing the double-exchange (DE) interaction, and hence favors the FM-metallic state over the COAFM state. As a result, the system behaves strong phase-separation when Ca is partially substituted by Sr. However, in the case of a full substitution (i.e. Pr_0.7_Sr_0.3_MnO_3_), the COAFM state and phase separation become trivial while the FM-metallic state due to DE interaction dominates the transport process^15^. The (011)-oriented PMN-PT single crystal was chosen as the substrate because of its perovskite-cubic structure (a_PMN-PT_ = 4.017 Å) and excellent anisotropic transverse piezoelectric effect^17^,^18^,^19^. The (011)-cut PMN-PT single-crystal slab behaves opposite piezoelectric behavior in the in-plane [100] and 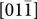 directions when an electric field is applied along the out-plane [011] crystalline direction, which could generate in-plane compressive stress along [100] and tensile stress along 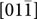
18 This strong in-plane anisotropic piezoelectric effect provides an exceptional opportunity for generating a large in-plane anisotropic strain in the epitaxially grown PSMO film on the PMN-PT substrate. Thus, a different electric-field-tuning magnetic memory effect due to the large in-plane anisotropic strains may be expected in the two in-plane directions.

In this study, by introducing an in-plane anisotropic strain field using the converse piezoelectric effect of the substrate, we observed an in-plane anisotropic, nonvolatile change of magnetization in the low-temperature FM states. More interestingly, these anisotropic modulations of magnetization persisted after the removal of the electric field (anisotropic strain-field) at low temperatures, indicating a nonvolatile magnetic memory effect in the wide-band PSMO film. Our analysis reveals that the preferential seeding and growth of FM domains, driven by the anisotropic strain field during the formation of FM ordering, lead to an induced-magnetic anisotropy in the FM film. After the electric filed is removed, this induced-anisotropy field results in a metastable magnetic state, which accounts for the nonvolatile memory effect in the wide-band PSMO film. Furthermore, we found that this anisotropic memory effect of ME coupling gradually disappears when the temperature approaches the metal-insulator transition, which can be ascribed to the collapse of the metastable magnetic state caused by increasing thermal energy. Our results clearly evidence that an electric-field-induced anisotropic strain can lead to a nonvolatile magnetic memory effect by forming a metastable magnetic state in a wide-band manganite, where phase separation is trivial. The competition between the barrier energy of the metastable magnetic state and the thermal energy is responsible for the observed temperature-dependent memory effect of ME coupling.

## Results

**Figure 1:**
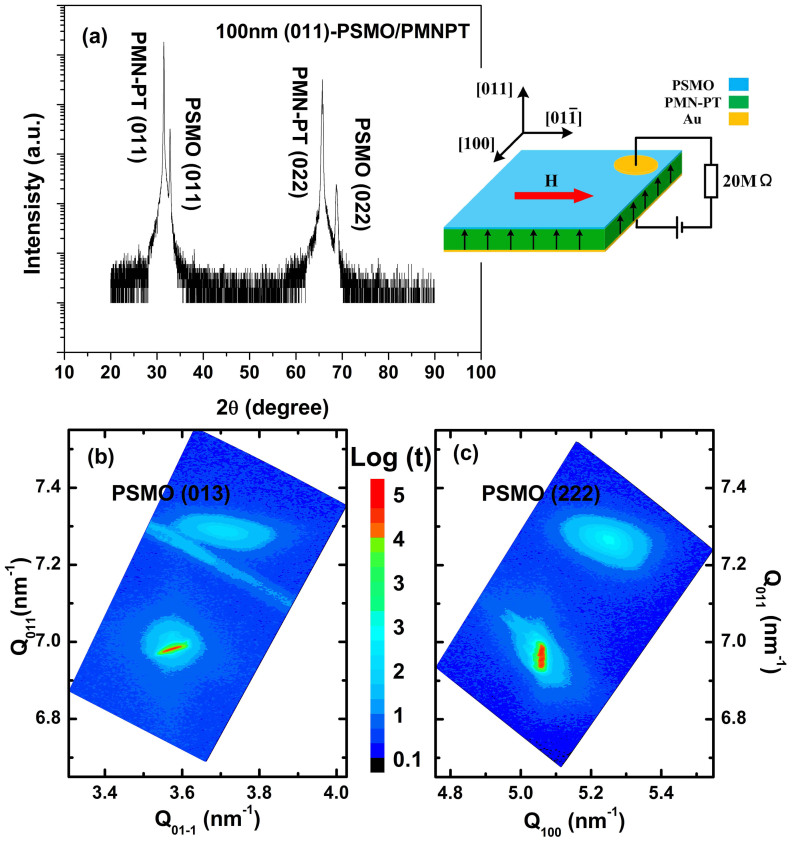
(a) X-ray diffraction patterns of the PSMO/PMN-PT heterostructure. X-ray reciprocal space maps around (b) (222) and (c) (013) reflections for the heterostructure. The inset in (a) shows the schematic circuit diagram for magnetic measurements under electric bias.

The out-of-plane interplanar distance and hence the out-of-plane epitaxial strain of the film were determined by means of x-ray diffraction (XRD) using Cu-Kα radiation. The XRD θ-2θ scan of the (011)-PSMO/PMN-PT heterostructure shows that the film is highly oriented along the [011] direction in the absence of any other impurity phases or textures (Fig. 1(a)). The out-of-plane interplanar distance was found to be ~2.727 Å, which is smaller than the bulk value^20^, indicating an out-of-plane compressive strain (~−0.3%). X-ray reciprocal space maps (RSMs) around asymmetric reflections were collected by a four-circle diffractometer to determine in-plane epitaxial strains of the film. Fig. 1(b) and (c) display the RSMs around (013) and (222) reflections. The extracted in-plane lattice constants of d_100_ and d_01-1_ for the film were 3.874 Å and 2.745 Å, respectively, which is slightly larger than those of bulk PSMO (3.857 and 2.735 Å, Ref. 20). The corresponding in-plane strains are tensile: 0.44% and 0.36% for [100] and 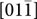 directions, respectively. We examined the performance of the PMN-PT substrate and found that applying an electric bias of ±6 kV/cm along [011] can induce a large compressive strain (~−0.31%) along in-plane [100] and a very small tensile strain (~0.018%) along in-plane 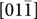 due to the large anisotropic piezoelectric effect of (011)-cut PMN-PT^21^, which can transfer to the overlying PSMO film^19^. The superimposed effect of lattice strains from both the epitaxial growth and the inverse piezoelectric effect cause the PSMO film to undergo a strong anisotropic tensile strain field along the two in-plane directions under ±6 kV/cm bias (i.e., ~0.13% for [100] and ~0.38% for 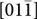). Under such in-plane anisotropic tensile strain, a considerable and different bending condition of Mn-O-Mn bonds along two in-plane directions is expected, which will cause anisotropic response in magnetic properties.

**Figure 2:**
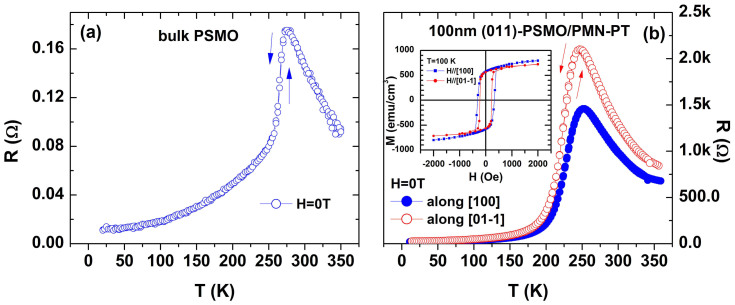
Temperature-dependent resistance of (a) bulk PSMO and (b) 100-nm-(011)-PSMO/PMN-PT film along in-plane [100] and 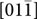 directions. The arrows indicate sweeping temperature direction. The inset in (b) displays magnetic hysteresis loops of PSMO film measured at 100 K for in-plane [100] and 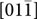 directions. The contributions from the substrate have been deducted.

We firstly measured the temperature-dependent resistance for PSMO bulk (Fig. 2 (a)) and 100-nm film (Fig. 2 (b)) on PMN-PT (along two perpendicular in-plane directions) in the absence of magnetic or electric fields. Fig. 2(a) shows that PSMO bulk exhibits zero thermal hysteresis around the metal-insulator transition, indicating that the double-exchange interaction dominates the transport process while phase separation behaves trivial, in accordance with previous reports^13^,^14^,^15^. Meanwhile, Fig. 2 (b) shows that the 100-nm film maintains the same features for both in-plane directions. However, the metal-insulator transition temperature (T_MI_) of the film decreased by ~24 K and ~29 K for in-plane [100] and 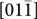 directions, respectively, in comparison with that in the bulk PSMO. This decrease can be attributed to an introduced epitaxial tensile strain in the film that could enhance the JT electron-phonon coupling and consequently favors the formation of a small polaron^10^,^11^. Furthermore, a small but distinct difference in the in-plane magnetic states is also observed in the two in-plane directions. The inset of Fig. 2(b) displays the magnetic hysteresis loops of the film measured along both in-plane directions at 100 K. Both the remnant magnetization (M_r_) and coercive field (H_C_) in the [100] direction are larger than those in the 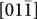 direction, indicating that magnetization prefers to align in the [100] direction. This magnetic anisotropy should be induced by the small anisotropic epitaxial strain^22^.

**Figure 3:**
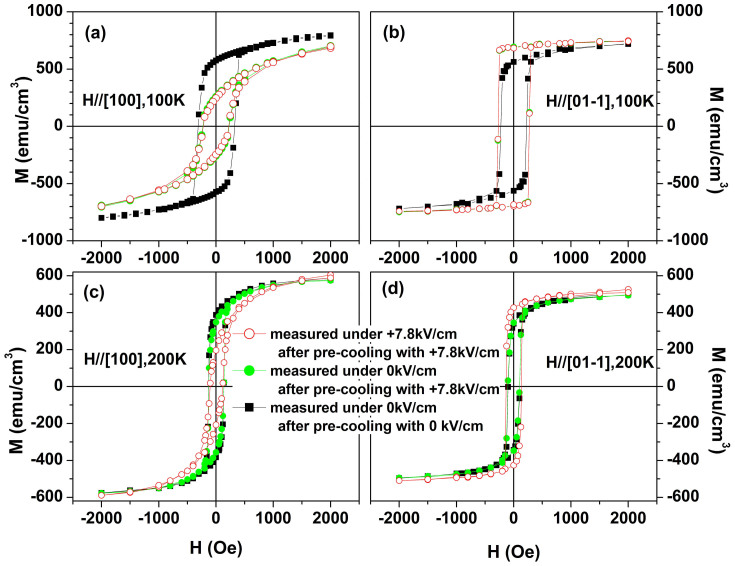
Magnetic hysteresis loops of PSMO film measured at the in-plane (a) [100] and (b) 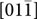 directions under electric fields +7.8 and 0 kV/cm at 100 K for the case of pre-cooling with a +7.8 kV/cm electric field and no magnetic field. (c) and (d) The corresponding loops at 200 K for the in-plane [100] and 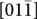 directions. For comparison, the loops measured under zero electric field for the case of pre-cooling without any electric or magnetic fields are also plotted.

The influence of the electric field, piezoelectric strain on magnetic hysteresis (M-H) loops of the film was further investigated for both in-plane [100] and 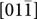 directions at different temperatures (Fig. 3). The samples were first cooled down to the target temperatures with a +7.8 kV/cm electric field and a zero magnetic field, then the M-H loops were measured under the same E-field (+7.8 kV/cm) (red curve) and zero E-field (green curve). To clearly demonstrate the effect of the strain or E-field, the M-H loops were also measured under zero electric field at the same temperatures after the sample was cooled under zero electrical and magnetic fields (black curve). Fig. 3(a) and (b) show the results measured at 100 K for [100] and 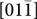 directions, respectively. Clearly, the modulation of magnetization behaves very differently under the same electric field for the two perpendicular in-plane directions at 100 K. In comparison with the zero field pre-cooling loop (black curve), the saturation magnetization, coercivity, and remanence of the +7.8 kV/cm electric field pre-cooling loop reduced significantly in the [100] direction. More importantly, the overall behavior of the loop also changed dramatically by becoming less square (red curve). Whereas for the 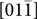 direction, the opposite trend was observed (the loop becomes more square). The modulation effect in magnetic properties can be partially reflected by the ratio of remnance, denoted as (M_r_(E)-M_r_(0))/M_r_(0), for both in-plane directions. Under a +7.8 kV/cm electric field, the ratios are about -54.9% and 23.3% for [100] and 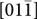, respectively. The coercivity in the two directions also behaves differently with an applied electric field (it decreases by 100Oe at [100] and increases by 46Oe at 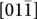, respectively). Such an anisotropic control of the magnetic properties is likely related to the efficient magnetic anisotropy induced by the electric bias. Considering that a relatively large tensile strain in the 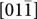 direction is induced by the applied electric field, as we mentioned above, to reduce the total energy, the FM domains prefer to grow along the 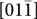 direction during the formation of magnetic ordering in the cooling process from the PM state. Therefore, the elongated FM-metallic domains are formed with magnetization along 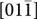
22,23, which generates an effective in-plane magnetic anisotropy and consequently reorients the easy axis towards the 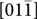 direction. This induced magnetic anisotropy increases both the remnant magnetization and coercivity in the 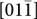 direction but reduces them in the [100] direction as shown in Fig. 3(a) and (b), respectively. It should be noted that the in-plane anisotropic strain field causes different bending of Mn-O-Mn bonds along the two in-plane directions. As a result, a strong overlap of the electronic clouds may occur in the direction with relatively large tensile strain (i.e. 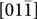), which will strengthen the FM double-exchange interaction along 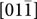 direction and contribute to the in-plane magnetic anisotropy.

Interestingly, the M-H loops behaved similarly after the removal of the electric field at 100 K for both in-plane directions (the green and red curves overlap). This observation indicates that the modulation of the magnetic properties by the electric-field-induced in-plane anisotropic strain is nonvolatile at 100 K, a temperature far below the T_MI_/T_C_ transition (see Fig. 2(b)). In other words, the film remembers the effect of the anisotropic strain on the magnetic properties and maintains the states even after the large in-plane anisotropic strain disappears along with the removal of the electric field. Such a nonvolatile modulation of magnetization in a wide-band manganite has not yet been reported and, therefore, deserves further investigation.

**Figure 4:**
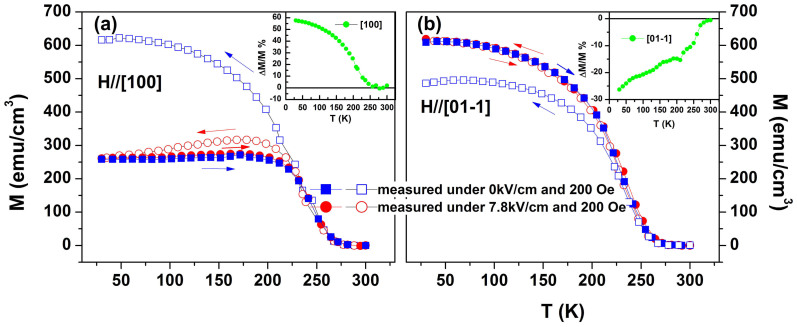
Temperature-dependent magnetization of PSMO film under a 0.02T magnetic field and +7.8 kVcm^−1^ (0 kVcm^−1^) electric field upon heating and cooling for the in-plane (a) [100] and (b) 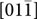 directions. Before measuring, the sample was cooled to 10 K with joint applications of both +7.8 kV/cm electric field and 0.02T magnetic field. The arrows indicate temperature sweeping directions. The insets in (a) and (b) present the temperature dependence of the relative magnetization change (ΔM/M(0) = (M(0)-M(E))/M(0)) between the cooling processes with +7.8 kV/cm and a zero electric field.

Fig. 3(c) and (d) show the M-H loops measured at 200 K approaching the T_MI_/T_C_. A similar but much less pronounced effect are observed in comparison with the data obtained at 100 K. With an applied electric field of +7.8 kV/cm, the ratio of (M_r_(E)-M_r_(0))/M_r_(0) are −41.9% and 22.7% for the [100] and 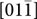 directions, respectively, whereas the coercivity decreases by 27Oe at [100] and increases by 32Oe at 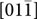. The less pronounced effect may be explained by the fact that at 200 K, the spins are not fully ordered ferromagnetically due to strong thermal agitation (see Fig. 4). More interestingly, the modulation of both the remnant magnetization and coercivity caused by the in-plane anisotropic strain disappears after the electric field is removed and hysteresis loops restore their original behavior—that measured at zero electric field upon pre-cooling without any electric or magnetic fields (green and black curves nearly coincide). This indicates that the electric field control on the magnetic properties at 200 K is volatile (i.e. the memory effect observed at low temperatures disappears as the temperature approaches T_MI_/T_C_). Such a temperature-dependent memory effect seems similar to that reported in the phase-separated (001)-Pr_0.6_Ca_0.4_MnO_3_/PMN-PT heterostructure^12^, in which the energy landscape of the phase-separation states plays a dominating role in the memory effect. However, here the wide-band PSMO film shows only trivial phase separation, indicating that an alternative mechanism must be invoked for the observed memory effect.

To better understand the role of temperature in electric field control of magnetic properties; in particular to explore the underlying mechanism of the memory effect in the strain-mediated ME coupling of the wide-band (011)-PSMO/PMN-PT heterostructure, we measured the temperature dependent magnetization (M-T) curves along both [100] and 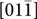 directions (Fig. 4 (a) and (b)). To this end, the sample was first cooled to 10 K in a +7.8 kV/cm electric field and a 0.02T magnetic field from room temperature, then the M-T curves were measured in the warming process and followed by the cooling process without changing either field (red curves in Fig. 4). Then, at 10 K, the electric field was removed and the magnetic field remained unchanged (0.02T), the M-T curves were measured again in the warming process and followed by the cooling process (blue curves in Fig. 4).

Several interesting features are apparent from Fig. 4. First, when the temperature was below the FM transition temperature (230 K), removing the electric field (+7.8 kV/cm) causes completely opposite changes to magnetization during the cooling process for two directions. The maximumal relative changes, defined by ΔM/M(0) = (M(0)-M(E))/M(0), were up to 57.7% and −26.3% in the [100] and 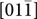 directions, respectively. Interestingly, the changes decrease with increasing temperature and vanish at the temperatures above the FM transition point, as shown in the insets to Fig. 4(a) and (b). These anisotropic magnetic responses to the external electric field are consistent with the results shown in Fig. 3 and should be ascribed to the in-plane anisotropic strain field induced by the electric field as discussed above. In fact, the cooling curves measured after the removal of the electric field are the same as the curves measured in a normally magnetic-field cooling process; this is because the magnetic state at 300 K becomes paramagnetic.

Second, during the heating process, the two M-T curves were nearly identical (red and blue curves) despite the application of an electric field (+7.8 or 0 kV/cm), providing more support for a nonvolatile control of magnetization by electric fields. Considering that the measurements of the both M-T curves are performed upon same pre-cooling process with joint applications of +7.8 kV/cm electric field and 0.02T magnetic field, this non-volatile control of magnetization can be certainly ascribed to the memory effect of the strain-mediated ME coupling^2^. It is important to note here that results from both Fig. 3 and 4 demonstrate that such a memory effect of ME coupling can only be observed in ferromagnically ordered states.

## Discussion

Previous studies have shown that in-plane strain can lead to local lattice distortions and make the large-scale electronic phase separation self-organize into elongated domains in the narrow-band phase-separated manganite system^22^,^24^. Specially, our recent studies on phase-separated Pr_0.7_(Ca_0.6_Sr_0.4_)_03_MnO_3_ films revealed that the in-plane anisotropic strain can be a driving force for the correlated occupation of metallic sites and the preferential formation of elongated FM metallic clusters, which leads to an abnormal thermal hysteresis^21^. Furthermore, studies based on the effect of electron-lattice coupling indicate that the in-plane strain field not only influences the formation of an electronic phase separation in narrow-band manganite films, but also couples to the spin and orbital degrees of freedom^22^,^25^ in wide-band manganite films, strongly affecting DE ferromagnetism. In addition, direct experimental observations have shown that the in-plane anisotropic stress from the lattice mismatch can lead to stripe domains in wide-band La_0.7_Sr_0.3_MnO_3_ films^23^.

**Figure 5:**
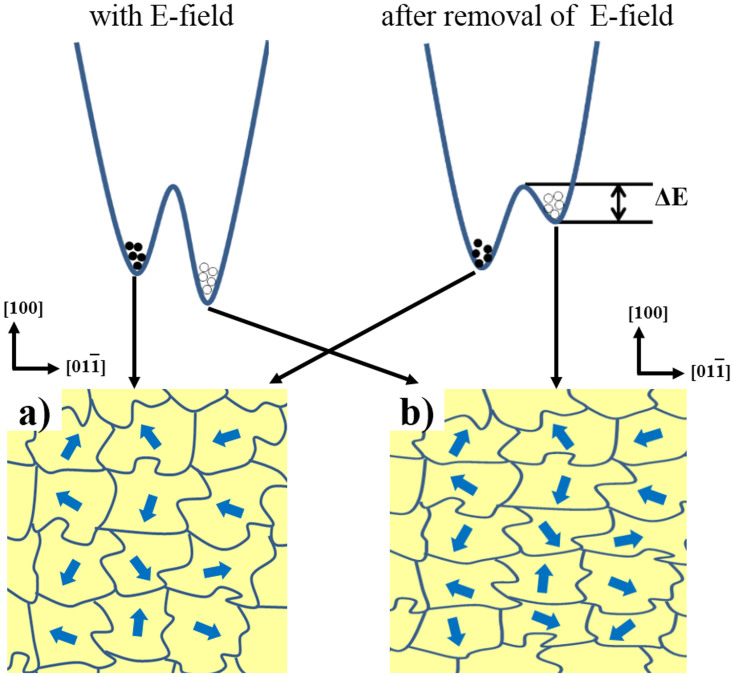
Schematic illustration of the FM-domain distributions in the PSMO film upon pre-cooling with (a) zero electric field (ground state) and (b) +7.8 kV/cm (metastable state). The upper part displays the schematic energy barrier between the metastable state and the ground state.

In the present wide-band (011)-PSMO/PMN-PT heterostructured system, the electric field induces a small tensile strain (~0.018%) along the 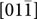 direction and a large compressive strain (~−0.31%) along the [100] direction. This anisotropic strain may then transfer to the PSMO film^18^,^19^ through the interface, which produces an anisotropic tensile strain field in the PSMO film. Such an anisotropic tensile strain-field may promote the distortion of the MnO_6_ octahedra and elongation of the Mn-O-Mn bond along the direction with a higher tensile strain field. As a result, the overlapping of electron clouds between adjacent atoms and the occupation of 3d orbitals appear preferential orientation, which appends an extra magnetic anisotropy to the double-exchange ferromagnetism. Accordingly, the FM domains in the PSMO film form and grow preferentially along the 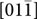 direction to reduce the energy required during the pre-cooling process with an electric field, consequently forming metastable magnetic states. Fig. 5(a) and (b) schematically illustrate the domain distribution in the PSMO film at ground and metastable states, respectively, showing how the FM domains grow preferentially along the 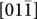 direction driven by the anisotropic strain field. With the electric-field tuning on, the state with the especially elongated FM domains has a lower energy than the ground state, making it thermodynamically stable (Fig. 5, upper left). Whereas removal of the applied pre-cooling electric field at 10 K causes the state to reach a higher local minimum energy (metastable) than the ground state (Fig. 5, upper right). These elongated domains in the metastable state bring about an additional magnetic anisotropy, which is responsible for the anisotropic modulation of magnetic behaviors of the film (Fig. 3 and 4). At low temperatures, the energy barrier (ΔE), produced by the shape anisotropic energy of elongate FM domains, blocked the system from recovering to a ground state at a zero electric field, due to the relatively small thermal energy in comparison with ΔE (Fig. 5, upper right). Until the amount of thermal energy is large enough to overcome the ΔE, as we observed in Fig. 3 (c) and (d), or to destroy the FM ordering, the material will continue staying in this metastable state. This explains why, below a certain temperature, the temperature-dependent magnetization of the film remains unchanged after the removal of the E-field and we observe the memory effect of ME coupling from anisotropic stain.

Previous researches investigating the strain-mediated magnetoelectric coupling in FM/FE heterostructures^6^,^12^,^26^ show that nonvolatile magnetic control was implemented by an electric-field induced remnant strain or an irreversible ferroelectric domain effect. However, a pre-established specific remnant strain state and a specific value or range of electric field to reset the strain are required to induce such effects in these systems^6^,^26^. Our results reveal that the nonvolatile magnetic memory effect could be simply realized by inducing a metastable magnetic state in the wide-band manganite layer using the electric-field induced anisotropic strain-field, demonstrating a new mechanism for the magnetoelectric memory effect. This unique magnetoelectric memory approach provides a promising technology for new design of spintronics devices such as the thermal-assisted electric-write magnetic memory.

## Methods

### Heterostructure fabrication

PSMO thin film was deposited on a (011)-oriented PMN-PT single crystal using the pulsed laser deposition (PLD) technique. The PSMO target was prepared by conventional solid reaction methods, and the commercial (011)-oriented PMN-PT single crystal was chosen as the substrate because of its perovskite-cubic structure (a_PMN-PT_ = 4.017 Å) and excellent anisotropic transverse piezoelectric effect. The strong in-plane anisotropic piezoelectric effect of PMN-PT provides an exceptional opportunity for generating a large in-plane anisotropic strain in the coherently grown PSMO film. During deposition, the temperature of the substrate was kept at 670°C and the oxygen pressure at 100 Pa. After deposition, the approximately 100-nm-thick film was cooled to room temperature in 1 atm of oxygen.

### Characterizations and measurements

The out-of-plane interplanar distance and hence the out-of-plane epitaxial strain of the film were determined by x-ray diffraction (XRD) using Cu-Kα radiation (Fig. 1(a)). In-plane epitaxial strains of the film were determined from X-ray reciprocal space maps (RSMs) around asymmetric reflections that were collected using a four-circle diffractometer (Bruker AXS D8-Discover). Au layers were vapor deposited on both the top and bottom of the PSMO/PMN-PT heterostructure as electrodes. The magnetic properties of the samples were measured using a superconducting quantum interference device (SQUID–MPMS) with *in situ* electric fields applied across the PSMO/PMN-PT structure by a Keithley 6517A electrometer. The leakage current was below 5nA under a 7.8 KV/cm electric field. A detailed circuit diagram is represented in the inset of Fig. 1(a).
